# Iatrogenic nerve injury following pelvic ring injury: a network meta-analysis

**DOI:** 10.1097/JS9.0000000000002272

**Published:** 2025-02-04

**Authors:** Yu-Cheng Su, Yung-Heng Hsu, Ying-Chao Chou, I-Jung Chen, Chih-Yang Lai, Yi-Hsun Yu

**Affiliations:** a Linkou Chang Gung Memorial Hospital, Taoyuan City, Taiwan; b Department of Orthopedic Surgery, Musculoskeletal Research Center, Chang Gung Memorial Hospital and Chang Gung University, Tao-Yuan, Taiwan

**Keywords:** closed reduction internal fixation, iatrogenic nerve injury, multi-trauma, pelvic ring injury, robotic-assisted technique

## Abstract

**Background:**

Pelvic ring injuries are common in multi-trauma patients and can be life-threatening, necessitating prompt surgical intervention to improve outcomes. However, surgery can lead to complications such as iatrogenic nerve injury. This network meta-analysis aimed to improve outcomes in multi-trauma patients with pelvic ring injuries by evaluating the incidence of iatrogenic nerve injuries, identifying vulnerable nerves, and comparing different fixation methods.

**Materials and methods:**

A systematic search of MEDLINE, EMBASE, and Scopus from inception to 5 December 2023 revealed 29 comparative studies on the incidence of iatrogenic nerve injury in 1561 adult patients with pelvic ring injuries. Data were extracted on study and patient characteristics, iatrogenic nerve injury incidences, and specific nerve injuries. A random-effects model assessed treatment effects, with subgroup analysis and meta-regression. The main outcomes included odds ratios (ORs) and confidence intervals (CIs) for iatrogenic nerve injuries.

**Results:**

Compared with closed reduction internal fixation, robotic-assisted techniques had the highest, and open reduction internal fixation had the lowest ORs for iatrogenic nerve injuries. The robotic-assisted approach ranked best with an OR of 0.22 (95% CI: 0.02–2.16), while closed reduction internal fixation with the anterior approach (OR: 0.71; 95% CI: 0.21–2.48) and open reduction internal fixation with the anterior approach performed the worst. The lateral femoral cutaneous nerve was injured in all open reduction internal fixation with anterior approach procedures and in 66.7% of open reduction internal fixation with posterior approach procedures. Meta-regression showed a significantly lower OR for iatrogenic nerve injuries in patients aged >41.4 years in the open reduction internal fixation with the anterior approach group (OR: 0.02; 95% CI: 0.001–0.63; *P* = 0.026) compared with younger patients.

**Conclusion:**

The robotic-assisted technique may result in the fewest iatrogenic nerve injuries during the treatment of pelvic ring injuries. The posterior approach may also reduce the risk of iatrogenic nerve injuries.

## Introduction

**Highlights**

- This network meta-analysis confirmed the superiority of certain approaches during the treatment of pelvic ring injuries.
- The robotic-assisted approach may involve the fewest iatrogenic nerve injuries.
- The posterior approach may reduce the risk of iatrogenic nerve injuries.
- Choosing an effective surgical approach is crucial for balancing treatment efficacy and complications.

Pelvic ring injuries (PRI) constitute 2–8% of fractures, affecting 20–25% of patients with multiple trauma^[1]^. These injuries vary from low-energy fractures associated with osteoporosis to high-energy disruptions in severe trauma^[1–3]^. Unstable injuries often require surgical stabilization to realign anatomy, and advances in understanding pelvic anatomy facilitate early intervention. Immediate operative management is crucial to improve prognosis and reduce complications and mortality^[2,3]^.

Operative management includes closed or open reduction and internal fixation with various constructs available^[4]^. Osteosynthesis corrects pelvic deformities and restores stability but can lead to complications^[4]^, including iatrogenic nerve injury (INI). INI may result from direct trauma during surgical procedures or fixator implantation and indirect injuries, such as stretch maneuvers during fracture reduction or ischemic effects from major vessel injuries^[5,6]^. INI following osteosynthesis for PRI can result in long-term sequelae and functional disability^[7,8]^.

Selecting the appropriate surgical intervention depends on clinical conditions and patient characteristics, including urgency, severity, and fracture pattern. Open reduction, using classical or newer approaches, focuses on safeguarding vital organs, vessels, and nerve structures during surgical dissection, fracture reduction, and fixator implantation^[4,9,10]^. However, altered anatomy after traumatic pelvic deformity, challenging access to deep pelvic spaces, and improper implant positioning can contribute to iatrogenic nerve injury (INI)^[5,10–12]^. Conversely, closed reduction with percutaneous fixation (using intramedullary screws or subcutaneous implants) is a minimally invasive alternative that minimizes tissue dissection^[7,8]^. To enhance precision, new minimally invasive techniques such as three-dimensional (3D) image-assisted and robotic-assisted (RA) approaches have been developed to ensure stable fixation while reducing the risk of neural or vascular damage^[13–18]^.

Existing studies have predominantly focused on surgical approaches, newly designed implants, and post-surgical radiological and functional outcomes. The actual relationship among the chosen surgical approach, applied fixation implants, INI incidence, and specific vulnerable nerves remains unclear. Therefore, we conducted a systematic review and network meta-analysis (NMA) to examine INI incidence, vulnerable nerves, and outcomes with different fixation methods and implants in PRI treatment.

## Materials and methods

### Ethics

#### Search methods for study identification

This study followed PRISMA (Preferred Reporting Items for Systematic Reviews and Meta-Analyses)^[19]^ and AMSTAR (Assessing the Methodological Quality of Systematic Reviews) guidelines^[20]^ (Supplemental Digital Content, Table 1, http://links.lww.com/JS9/D873) and registered on PROSPERO. We searched MEDLINE, EMBASE, and Web of Science for clinical studies on fixation methods for PRIs from inception to 5 December 2023, without language restrictions. Additional studies were identified through reference lists. The search strategy details are in Supplemental Digital Content (Table 2, http://links.lww.com/JS9/D873).

#### Inclusion and exclusion criteria

This review included prospective and retrospective comparative studies (evidence levels 2 to 3), primarily investigating INI incidence in adult patients with PRI. It examined various operative treatments, including the RA approach, open reduction internal fixation with the anterior (ORIF-A) or posterior approach (ORIF-P), and closed reduction internal fixation with the anterior (CRIF-A) or posterior approach (CRIF-P). Regarding CRIF treatment options, minimally invasive percutaneous fixation, anterior subcutaneous internal fixator (ASIF), and external fixation were included. Three-dimensional image-assisted techniques comprised a “3D-navigated group” and a “3D-printing group,” defined as “intraoperative 3D image navigation for percutaneous screw fixation” and “preoperative preparing on a 3D-printed model for patient-specific intervention,” respectively. The RA group was defined as using a robotic guidance system for fixation. Groups were determined as the anterior or posterior group depending on the main title or dominant approach used in the selected studies. Pediatric trials, cadaveric studies, and studies with unknown/incomplete/inappropriate outcomes, duplicate data, stress/open/pathologic fractures, and unclear implant usage or outcome measurements were excluded. The excluded articles are listed in Supplemental Digital Content (Table 3, http://links.lww.com/JS9/D873).

#### Measurement of outcomes and treatment effects

We examined two main outcomes: the incidence of INI across different fixation approaches and specific nerve injury rates associated with various surgical treatments. Nerve injuries assessed included the lateral femoral cutaneous nerve (LFCN), femoral nerve, fifth lumbar spinal nerve (L5), first sacral nerve (S1), lumbosacral plexus injuries, and sacral plexus injuries. Treatment effects were assessed using odds ratios (ORs) and confidence intervals (CIs) for categorical variables.

#### Data collection and analysis

1. Selection of studies

One independent author reviewed titles and abstracts and conducted full-text evaluations. In case of disagreements, a senior co-author was consulted to reach a consensus.

2. Data extraction and handling missing data

A single author independently extracted data on the first author, publication year, study design, inclusion/exclusion criteria, patient characteristics, patient numbers, fracture type, implant choice, fixation method, and iatrogenic nerve injuries. Collected data included patient age, sex, mean follow-up duration, publication year, study type, and fracture type definitions. No missing data required adjustments. A senior author verified the data extraction process.

3. Risk of bias assessment

One evaluator independently assessed methodological quality using the risk of bias in non-randomized studies of interventions (ROBINS-I)^[21]^ instrument for non-randomized trials and version 2 of the Cochrane risk-of-bias tool (RoB 2)^[22]^ for randomized trials. In case of disagreement, a second author was consulted. A summary of the bias risk is provided in Supplemental Digital Content (Table 4, http://links.lww.com/JS9/D873).

4. Data synthesis

Statistical analyses for NMA were conducted using STATA (StataCorp. 2017. Stata Statistical Software: Release 15; StataCorp LP, College Station, TX). Traditional pairwise meta-analysis enabled direct comparisons between treatments, whereas NMA integrated both direct and indirect evidence to improve the estimation of treatment effects across a network of interventions, especially when direct comparisons were limited or absent. Random-effect models were employed for all outcomes. Heterogeneity was assessed using *I*^2^ and Cochrane Q tests. NMA compared multiple interventions and assessed consistency using design-by-treatment, loop inconsistency, and node-splitting models. Subgroup analyses used meta-regression to explore patient characteristics such as age, sex (female or male dominant), publication type (prospective or retrospective), publication year, and fracture severity (partially stable dominant, including Tile B and Arbeitsgemeinschaft für Osteosynthesefragen /Orthopedic Trauma Association [AO/OTA] 61B fracture classification; and unstable dominant, including Tile C and AO/OTA 61C fracture classification). The surface under the cumulative ranking curve (SUCRA) was used to rank the comparative efficacy of treatments, and publication bias was evaluated using funnel plots and Egger’s regression. The quality of evidence from this NMA was assessed using the Grading of Recommendations Assessment, Development, and Evaluation (GRADE) tool^[23,24]^.

## Results

### Study selection and description

**Figure 1.**
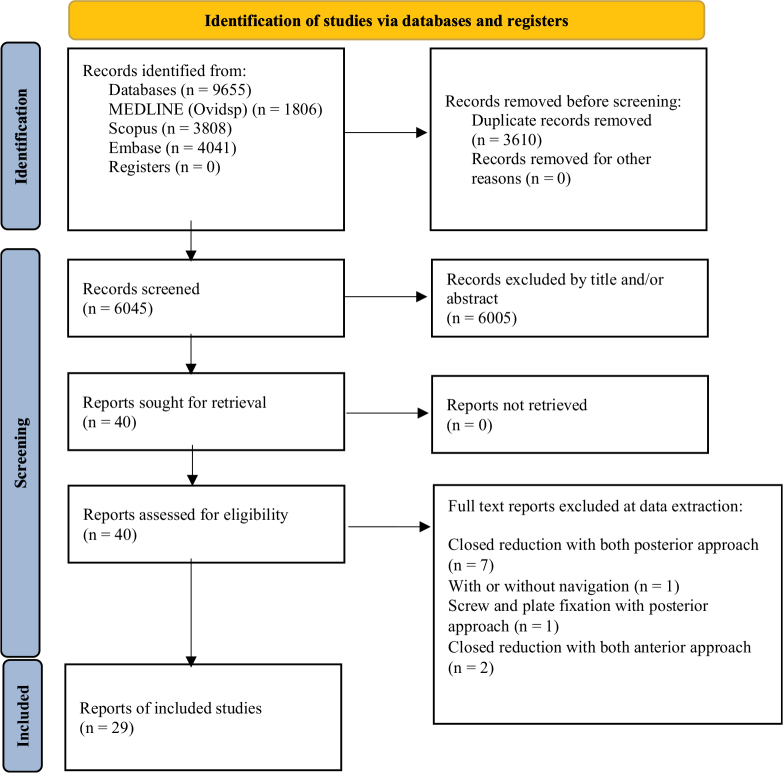
PRISMA flow diagram for the network meta-analyses.

**Figure 2.**
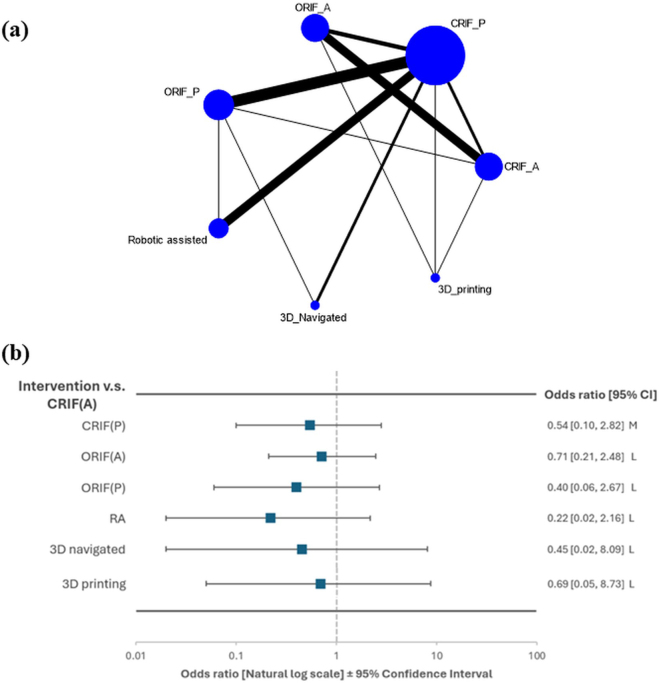
The result of network meta-analyses with (a) network geometry and (b) forest plot of meta-analysis results with confidence ratings for iatrogenic nerve injuries. CI, confidence interval; L, low confidence rating; M, moderate confidence rating; CRIF-A, closed reduction internal fixation with the anterior approach; CRIF-P, closed reduction internal fixation with the posterior approach; ORIF-A, open reduction internal fixation with the anterior approach; ORIF-P, open reduction internal fixation with the posterior approach; 3D-navigated, three-dimensional navigated approach; 3D printing, three-dimensional printing-assisted approach; RA, robotic-assisted approach.

This analysis included 29 (six prospective and 23 retrospective) studies involving 1561 patients (Table 1 and Fig. 1). The trials included the following interventions: RA approach (six trials)^[13-15,25–27]^, 3D-navigated approach (three)^[13,15,26]^, 3D-printing approach (three)^[14,26,27]^, ORIF-A approach (nine)^[4,9,16,26,28–34]^, ORIF-A approach (10)^[10,18,25,28,35–40]^, CRIF-A (nine)^[4,9,14,28,29,32–34,37,41]^, and CRIF-P approach (20)^[4,10,13,15–18,27,28,30,31,35,36,38–44]^. All interventions were compared directly and indirectly, presented in network geometry (Fig. 2).

**Table 1:** Characteristics of the included trials

No.	Author	Publication year	Nation	Study type	Sample size	Sex (M/F)	Age (mean)	Fracture type	F/U (months)	Group	Treatment details	INI (*N*)
1	Suzuki	2007	Japan	Retrospective	22	28;12	42.4	Tile B/C = 39/18	N/A	CRIF (ant)	External fixation (definitive)	0
	Suzuki	2007	Japan	Retrospective	12	As above	As above	Tile B/C = 39/18	N/A	ORIF (post)	ORIF (post)	0
2	Hiesterman	2012	US	Surgical technique/randomized controlled trial	5	4;7	N/A	N/A	5.1 months	CRIF (ant)	External fixation	0
	Hiesterman	2012	US	Surgical technique/randomized, controlled trial	6	4;7	N/A	N/A	5.1 months	ORIF (ant)	APIF reconstruction plate	0
3	Liu	2014	China	Retrospective	23	16;7	18–65	Tile B/C = 8/15	N/A	CRIF (ant)	Small-window minimally invasive surgery	0
	Liu	2014	China	Retrospective	38	26;12	18–65	Tile B/C = 13/25	N/A	ORIF (ant)	Conventional ilioinguinal treatment	0
4	Vaidya	2016	US	Retrospective	24	18;6	43.38 ± 18.13	APC = 7; LC = 4; V = 13	40.44 months	CRIF (ant)	ASIF, * 9 EXFIX placed prior to INFIX	1
	Vaidya	2016	US	Retrospective	28	25;3	43.38 ± 18.13		40.44 months	ORIF (ant)	plate	0
5	Gu	2017	China	Retrospective	26	16;10	39.4 ± 10.8	Tile B/C = 23/3	1 year	CRIF (ant)	Sacroiliac anterior papilionaceous plate	1
	Gu	2017	China	Retrospective	26	17;9	37.5 ± 11.4	Tile B/C = 22/4	1 year	ORI F (ant)	Sacroiliac anterior plate fixation	6
	Gu	2017	China	Retrospective	26	18;8	38.7 ± 13.5	Tile B/C = 23/3	1 year	CRIF (post)	Percutaneous sacroiliac screw internal fixation	0
6	Wang	2017	China		26	16	39.3 ± 17	Tile B1/2/3 = 8/13/5	8.2	CRIF (ant)	ASIF	2
	Wang	2017	China	Retrospective	26	15	38.2 ± 13.1	Tile B1/2/3 = 4/15/7	9.3 mess	ORIF (A)	Plate and screws	0
7	Tsai	2019	China	Retrospective	7	2	36.00 ± 7.33	Tile B/C = 5/2	25.3	CRIF (ant)	Minimal invasive screw	0
	Tsai	2019	China	Retrospective	7	4	44.71 ± 20.00	Tile B/C = 4/3	25.3	CRIF (ant)	ORIF (Plate)	0
8	Yin	2019	China	Retrospective	35	19;16	41.7 ± 12.6	AO/OTA 61B/C = 25/10	27	CRIF (ant)	ASIF	10
	Yin	2019	China	Retrospective	39	24;15	43.6 ± 13.2	AO/OTA 61B/C = 25/14	23	ORIF (ant)	Plate fixation	0
9	Zhou	2022	China	Retrospective	8	6; 2	52.5 (16.9)	Tile B1/B2.1/B3 = 2/4/2	13.25	CRIF (ant)	External fixation	0
	Zhou	2022	China	Retrospective	10	7;3	45.2 (9.9)	Tile B1/B2.1/B3 = 3/4/3	13.25	3D printing	3D printing template	0
10	Wang	2022	China	Retrospective	36	19;17	38.1 (13.5)	LC-1 pelvic fracture (OTA 61-B2.1) with a complete sacral fracture	29.3 (5.9)	CRIF (ant)	EXFIX or screw fixation	1
	Wang	2022	China	Retrospective	32	21;11	40.8 (15.5)	LC-1 pelvic fracture (OTA 61-B2.1) with a complete sacral fracture	29.3 (5.9)	CRIF (post)	Percutaneous sacroiliac joint screws	1
11	Templeman	1996	US	Retrospective	13	*21; 9	36	Displaced fractures of the sacrum	28	CRIF (post)	Percutaneous	1
	Templeman	1996	US	Retrospective	17	*21; 9	36	Displaced fractures of the sacrum	28	ORIF (post)	Screw fixation	1
12	Wu	2015	China	Retrospective	20	11;9	39.5 (13.2)	AO/OTA 61-type B1/B2/B3 = 4/4/2; C1/C2/C3 = 6/3/1	27.3	CRIF (post)	Minimally invasive adjustable plate	0
	Wu	2015	China	Retrospective	24	13;11	34.3 (10.1)	AO/OTA 61-type B1/B2/B3 = 8/6/2; C1/C2 = 5/3	21.8	ORIF (post)	Locking compression plate	0
13	Li	2015	China	Retrospective	38	21;17	35.2 (10.9)	Tile B1/B2/B3 = 7//7/4; C1/C2/C3 = 9/6/5	12.7 months	CRIF (post)	Percutaneous iliosacral screws + conventional C-arm guided	0
	Li	2015	China	Retrospective	43	24;19	36.4 (11.9)	Tile B1/B2/B3 = 8/8/5; C1/C2/C3 = 9/7/6	12.7 months	3D-navigated	Percutaneous iliosacral screws + 3D C-arm fluoroscopy navigation	0
14	Li	2018	China	Retrospective	7	5;2	33.6 ± 11.4	Tile type B/C = 6/1	12 months	CRIF (post)	Percutaneous sacroiliac joint screw fixation	3
	Li	2018	China	Retrospective	13	8;5	35.7 ± 12.3	Tile Type B/C = 7/6	12 months	ORIF (post)	Posterior tension belt plate fixation	2
15	Yang	2018	China	Retrospective	18	10;8	50.1 (13.7)	AO/OTA 54B/54C = 4/14	N/A	3D printing	3D external template-guided percutaneous iliosacral screw	0
	Yang	2018	China	Retrospective	22	11;11	51.7 (15.2)	AO/OTA 54B/54C = 7/15	N/A	CRIF (post)	Conventional fluoroscopy-guided percutaneous iliosacral screw	0
16	Long	2019	China	Retrospective	35	21;14	35.68 (8.37)	Tile type B/C = 15/20	8 to 32 months	CRIF (post)	Percutaneous screw fixation	0
	Long	2019	China	Retrospective	56	32;24	35.95 (7.75)	Tile type B/C = 21/35	8 to 32 months	Robotic-assisted (RA)	Robotic assisted (RA)	0
17	Hung	2019	Taiwan	Retrospective	16	10;6	35.44 (13.52)	Tile type A/B/C = 1/6/9	24.3	3D printing	3D printing-assisted contoured plate	0
	Hung	2019	Taiwan	Retrospective	14	8; 6	35.64 (17.37)	Tile type A/B/C = 1/8/5	24.3	ORIF (ant)	Conventional locking plate fixation	0
18	Abou-Khalil	2020	Switzerland	Retrospective	14	12;2	42.5 (26; 54)	Tile type B/C = 6/8	10.5	ORIF (post)	Screw and plate fixation	1
	Abou-Khalil	2020	Switzerland	Retrospective	22	18;4	35.5 (27; 59)	Tile type B/C = 3/19	6	CRIF (post)	Percutaneous iliosacral screw fixation	1
19	Hoffmann	2021	Germany	Retrospective	31	18;13	56.6 (18.7)	OTA/AO 61type C	N/A	3D-navigated	Navigated screw fixation	0
	Hoffmann	2021	Germany	Retrospective	32	19;12	59.9 (18.4)	OTA/AO 61type C	N/A	ORIF (post)	Standard screw fixation	0
20	Li	2022	China	Retrospective	48	35;13	41.5 (7.3)	Tile type B1/B2/B3 = 8/7/6; C1/C2/C3 = 9/8/10	N/A	CRIF (post)	Percutaneous screw fixation	0
	Li	2022	China	Retrospective	47	33;14	40.3 (8.2)	Tile type B1/B2/B3 = 6/8/9; C1/C2/C3 = 6/10/8	N/A	Robotic-assisted (RA)	Robot-assisted percutaneous screw fixation	0
21	Li	2023	China	Retrospective	27	16;11	53.78 ± 7.470	Tile type B/C = 14/13	N/A	CRIF (post)	Percutaneous screw fixation	0
	Li	2023	China	Retrospective	30	21;9	57.33 ± 9.211	Tile type B/C = 12/18	N/A	Robotic-assisted (RA)	Robot-assisted percutaneous screw fixation	0
22	Li	2014	China	Prospective	32	25;7	37.3 (16.5)	Tile type C1/C2/C3 = 23/8/1	N/A	ORIF (ant)	Sacroiliac anterior plate fixation + screw internal fixation	**0**
	Li	2014	China	Prospective	32	24;8	39.3 (18.4)	Tile type C1/C2/C3 = 22/8/2	N/A	CRIF (post)	Percutaneous sacroiliac screw internal fixation	**0**
23	Yin	2014	China	Retrospective	26	15;11	39.58	All tile type C	14.11 (1.94)	CRIF (post)	Iliosacral screw fixation	**0**
	Yin	2014	China	Retrospective	37	21;16	37.62	All tile type C	14.11 (1.94)	ORIF (post)	Reconstruction plate fixation	**0**
24	Herman	2016	US	Retrospective	7	36;54	36.1	Denis zone II	N/A	ORIF (post)	Plate	**0**
	Herman	2016	US	Retrospective	83	36;54	36.1	Denis zone II	N/A	CRIF (post)	SI screw	**0**
25	Elzohairy	2017	Egypt	Retrospective	35	25;10	N/A	Tile type B1/B2/B3 = 13/8/5; C1/C2 = 5/4; Day type III = 25	N/A	CRIF (post)	Iliosacral screw fixation	**0**
	Elzohairy	2017	Egypt	Retrospective	35	28;7	N/A	Tile type B1/B2/B3 = 10/12/4; C1/C2 = 6/3; Day type I/II = 8/18	N/A	ORIF (post)	Plate fixation	**0**
26	Liu	2018	China	Retrospective	21	13;8	39.8 (7.1)	Tile type B/C = 11/3	5.4	CRIF (post)	Percutaneous sacroiliac iliosacral screw fixation	**0**
	Liu	2018	China	Retrospective	24	15;9	37.4 (6.6)	Tile type B/C = 10/6	5.4	Robotic assisted (RA)	Robotic assisted (RA)	**0**
27	Zhu	2023	China	Retrospective	45	22;23	47.6 (13.9)	Tile Type B1/B2/B3 = 3/38/4	20.3 (10.4)	ORIF (post)	Ilioiliac plate/spino-pelvic fixation	**0**
	Zhu	2023	China	Retrospective	10	4;6	53.3 (16.4)	Tile Type B1/B2/B3 = 2/7/1	18.4 (5.6)	CRIF (post)	Percutaneous screw fixation	**0**
	Zhu	2023	China	Retrospective	40	21;19	49.0 (15.0)	Tile type B1/B2/B3 = 1/37/2	16.5 (3.8)	Robotic assisted (RA)	Percutaneous screw fixation	**0**
28	Pei	2023	China	Retrospective	35	16;19	44.28 (14.94)	Tile type B2/B3 = 6/4; C1/C2/C3 = 11/5/9	14.42 (1.57)	3D-navigated	Percutaneous IS screw fixation, 3D-navigated	**0**
	Pei	2023	China	Retrospective	29	15;14	39.89 (12.78)	Tile type B2/B3 = 3/4; C1/C2/C3 = 14/6/2	14.42 (1.57)	CRIF (post)	Minimally invasive percutaneous plate fixation	**0**
29	Gilani	2023	US	Prospective	10	7;3	50.8 (14.77)	N/A	N/A	Robotic-assisted (RA)	Percutaneous IS and TS screw	**0**
	Gilani	2023	US	Prospective	11	8;3	38.4 (15.8)	N/A	N/A	CRIF (post)	Percutaneous IS and TS screw	**0**

F/U, follow-up period; INI, iatrogenic nerve injury; CRIF-A, closed reduction internal fixation with the anterior approach; CRIF-P, closed reduction internal fixation with the posterior approach; ORIF-A, open reduction internal fixation with the anterior approach; ORIF-P, open reduction internal fixation with the anterior approach; 3D-navigated, three-dimensional navigated approach; 3D printing, three-dimensional printing-assisted approach; RA, robotic-assisted approach; O-B, open-book; LC, lateral compression; RV, rotationally and vertically; YB, Young and Burgess classification; LC, lateral compression; APIF, subcutaneous anterior pelvic internal fixator; APC, anterior posterior compression fracture; ASIF, anterior subcutaneous internal fixator; VS, vertically stress; CM, combined mechanism; AO/OTA, The AO Foundation/Orthopedic Trauma Association (AO/OTA) fracture classification.

^*^Data are presented as ratios and numbers.

### Primary outcomes

The results of the NMA, including odds ratios (ORs) with 95% confidence intervals (CIs), are shown in Supplemental Digital Content (Figure 1 http://links.lww.com/JS9/D874 and Table 5 http://links.lww.com/JS9/D873). Compared with the control (CRIF-A), RA and ORIF-A had the highest and lowest combined odd ratios of 0.22 (95% CI: 0.02, 2.16) and 0.71 (95% CI: 0.21, 2.48) for INIs among various interventions for PRIs, respectively (Supplemental Digital Content, Figure 1, http://links.lww.com/JS9/D874 and Table 5, http://links.lww.com/JS9/D873).

**Figure 3.**
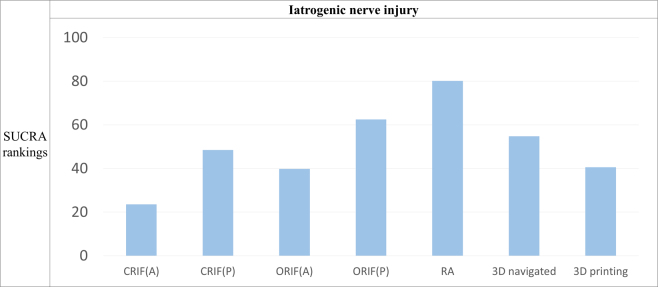
Relative ranking probability of various pelvic ring injury fixation methods for iatrogenic nerve injuries. CRIF-A, closed reduction internal fixation with the anterior approach; CRIF-P, closed reduction internal fixation with the posterior approach; ORIF-A, open reduction internal fixation with the anterior approach; ORIF-P, open reduction internal fixation with the anterior approach; RA, robotic-assisted approach; 3D-navigated, three-dimensional navigated approach; 3D printing, three-dimensional printing-assisted approach; SUCRA, surface under the cumulative ranking curve.

Based on the SUCRA scores (Fig. 3), the RA approach (SUCRA = 80.2%) ranked the best for the lowest odd ratios (0.22; 95% CI: 0.02, 2.16) of INIs, whereas CRIF-A (SUCRA = 23.6%) and ORIF-A (SUCRA = 39.8%) performed the worst.

Regarding the distribution of distinct INIs, LFCN injury was the most vulnerable to INI with the anterior approach, with all occurring in ORIF-A (15/15) and 66.7% of ORIF-A (4/6). CRIF-P injuries mainly involved the fifth lumbar spinal nerve (L5; 2/7) and lumbosacral plexus (3/7) (Table 2).

**Table 2:** Ratio of distinct iatrogenic nerve injuries across various interventions for pelvic ring injuries

Intervention	Studies (*N*)	Sample size (*N*)	INI (*N*)	LFCN	FCN	L5	L/S	Unspecified
CRIF-A	9	205	15	15	0	0	0	0
CRIF-P	20	523	7	2	0	2	3	0
ORIF-A	9	216	6	4	0	0	2	0
ORIF-P	10	236	4	0	0	1	0	3
RA	6	207	0	0	0	0	0	0
3D-navigated	3	44	0	0	0	0	0	0
3D printing	3	109	0	0	0	0	0	0

CRIF-A, closed reduction internal fixation with the anterior approach; CRIF-P, closed reduction internal fixation with the posterior approach; ORIF-A, open reduction internal fixation with the anterior approach; ORIF-P, open reduction internal fixation with the anterior approach; RA, robotic-assisted approach; 3D-navigated, three-dimensional navigated approach; 3D printing, three-dimensional printing-assisted approach; INI, iatrogenic nerve injury; LFCN, lateral femoral cutaneous nerve; FCN, femoral nerve; L5, the fifth lumbar nerve; L/S, lumbar or sacral nerve plexus. Data are presented as integral numbers.

### Sensitivity analysis

The meta-regression with publication year, publication type, injury type, and sex did not moderate INI outcomes (Supplemental Digital Content, Table 6, http://links.lww.com/JS9/D873). Nonetheless, the ORIF-A group (OR, 0.02; 95% CI 0.001–0.63; *P* = 0.026) among older patients (cutoff value was 41.4 years) had a significantly lower OR for INI than that of younger patients (Supplemental Digital Content, Figure 2, http://links.lww.com/JS9/D875)

### Reporting bias

The outcomes indicated low publication bias likelihood, with Egger’s regression showing no significant asymmetry (*P* = 0.308) (Supplemental Digital Content, Figure 3, http://links.lww.com/JS9/D876). The GRADE confidence rating was low to moderate for all outcomes (Supplemental Digital Content, Table 7, http://links.lww.com/JS9/D873).

### Assessment of inconsistencies

The outcomes showed no significant global inconsistency (*P* = 0.2853; Supplemental Digital Content, Table 8, http://links.lww.com/JS9/D873). Despite significant local inconsistency via side-splitting (*P* = 0.012), the loop approach found no global inconsistency (*P* = 0.9769).

## Discussion

This represents the first NMA investigating INI incidence in PRIs across diverse surgical approaches. The RA technique exhibited the lowest OR for INIs, emerging as the most promising strategy for reducing INI incidence. Notably, all INIs observed in the CRIF-A group were LFCN injuries. Anterior approaches, using ORIF or CRIF, appeared less favorable and displayed higher odds of INIs. 3D-navigated approaches showed potential, outperforming the anterior approach but falling short of the efficacy observed with the anterior approach. These findings underscore the significance of selecting a surgical approach to minimize INI risk, and RA might be the preferred option.

Our NMA emphasizes exploring innovative surgical methods to reduce nerve injury incidence. Successful outcomes following osteosynthesis for PRI rely on achieving anatomical reduction and secure fixation, prompting the introduction of new operative techniques over time. Open reduction using traditional and novel surgical techniques prioritizes safeguarding adjacent vital organs, blood vessels, and nerves. Yu *et al* adapted the pararectus approach, initially introduced by Keel *et al*, for complex acetabular fractures in treating PRI fixation^[45,46]^. Our study did not report specific INIs affecting these nerves, although the obturator nerve may be at risk with this approach.

Newly modified minimally invasive techniques offer advantages such as reduced surgical invasiveness, less radiation exposure, improved technical safety, and procedural efficiency^[4,9,30,39]^. Consequently, they provide effective supplementary options for treating unstable PRI cases. Our analysis indicates that open reduction via the anterior approach correlates with a higher complication incidence compared with the posterior approach. The LFCN was vulnerable in approximately two-thirds of ORIF-A cases. Additionally, injuries to the L5 nerve were observed, although specific details were often unspecified.

The use of large cannulated screws and improved fluoroscopic imaging has popularized iliosacral screw placement from the lateral ilium across the sacroiliac joint into the upper sacral vertebral body for posterior pelvic fixation^[47]^. However, percutaneous fixation for posterior PRI may lead to complications such as fixation failures, screw misplacement, nerve injuries, infections, and suboptimal reductions^[8,10,38]^. In our study, injuries to the L5 nerve and the lumbar/sacral nerve plexus predominated, accounting for over 70% of cases in the CRIF-P group. For anterior PRI, the INFIX construct offers a novel approach using standard spinal hardware components to create a subcutaneous pelvic fixator, aiming to avoid complications associated with external fixation, particularly LFCN injury^[32]^.

Intraoperative 3D computed tomography images guide percutaneous screw insertion, and surgeons can prepare for surgical pelvic reconstruction using fixation plates based on a 3D-printed model preoperatively^[13–15,25–27]^. Traditional surgical techniques involve fully exposing the fracture and contouring the implant on the spot, increasing invasiveness, tissue damage, bleeding, and surgery times. Precontoured plates offer minimal invasiveness, reducing soft tissue dissection and detachment. Consequently, it may significantly reduce the risk of intraoperative LFCN injuries^[26]^. To improve the accuracy of screw fixation, CT-guided insertion and a robotic technique were adapted in PRI^[16–18,42–44]^.

RA techniques improve screw fixation accuracy in PRIs by ensuring precise positioning and stable insertion paths, minimizing cortical penetration. Real-time optical tracking reduces operation times and radiation exposure^[16,42–44]^, notably decreasing the risk of inadvertent damage to nearby nerves and vital structures, which lowers postoperative complications and enhances patient recovery outcomes^[43,44]^. In our analysis, RA systems demonstrated a lower incidence of INI compared with traditional surgical methods. RA effectively reduced unplanned cortical or neuroforaminal violations, common sources of iatrogenic damage to the lumbosacral plexus during pelvic surgeries^[17]^. The RA system reduced surgery duration by approximately 25–50% compared with conventional techniques^[16,42–44]^. Additionally, RA fixation significantly reduced the frequency of fluoroscopy by 44–72%^[16,44]^. In terms of intraoperative bleeding, RA fixation was associated with a decrease in total blood loss ranging from 21% to 24%^[16,44]^. Compared with traditional methods, precise stabilization with RA fixation leads to improved recovery and satisfactory functional outcomes^[18,43,44]^.

INIs significantly impair function, mobility, and quality of life in patients with PRIs^[7,8]^. These injuries often involve specific nerves located in the pelvic region. For instance, the LFCN, which passes through the inguinal ligament and supplies sensation to the lateral thigh, can be damaged directly by cutting, implant-related injury, indirect stretching, or dissection^[5]^. Damage to the LFCN leads to meralgia paresthetica, characterized by burning, tingling, or numbness in the outer thigh. Our findings align with previous studies indicating that LFCN irritation is a common complication associated with ASIF application^[32,34]^. Takeda *et al* investigated the impact of nerve-to-implant (INFIX) distance on postoperative LFCN symptoms and found that distances ≥13 mm significantly reduced the risk of nerve disorders. In contrast, shorter distances or unclear nerve identification during surgery increased complications^[11]^. Similarly, the femoral nerve, originating from the lumbar plexus and passing beneath the inguinal ligament through the pelvis, innervates anterior thigh muscles and provides sensory function to the thigh and medial leg. Damage to the femoral nerve can result in weakness in hip flexion and knee extension, impacting activities such as walking and stair climbing. Hesse *et al* highlighted the serious risk posed to femoral nerves when using INFIX for PRI treatment^[48]^. Even with immediate implant removal upon detecting nerve damage, some patients experienced persistent weakness in the quadriceps, altered skin sensation in the thigh, and gait abnormalities due to femoral nerve palsy during initial follow-up^[48]^.

From the posterior direction, the L5 nerve root, originating from the lumbar spine and extending through the pelvis, contributes to motor function and sensation in the lower extremities. Damage to the L5 nerve can cause weakness in ankle dorsiflexion and sensory deficits along the lateral leg and dorsum of the foot, impacting walking and gait mechanics. Our NMA findings align with previous studies that reported a significant drawback of percutaneous screw posterior fixation due to its proximity to the lumbosacral plexus, particularly L5^[10,16,41]^. Furthermore, the sacral nerves, including the sciatic nerve, exit the pelvis through the sciatic notch and provide motor function and sensation to the posterior thigh, leg, and foot. Injury to these nerves can lead to weakness in hip extension and knee flexion and sensory deficits in the posterior thigh, leg, and foot, affecting mobility and balance during various activities^[10,16]^.

In our meta-regression subgroup analysis, we found a significant decrease in INIs within the ORIF-A group when comparing older patients to younger ones, using an age threshold of 41.4 years. This suggests that older patients in the ORIF-A group experienced fewer INIs than younger patients. Mann *et al* reported an average age of 46 years for high-energy trauma patients in Canada, highlighting that younger patients typically sustain these injuries through high-energy impacts, whereas older individuals experience them through low-energy impacts such as falls, often complicated by osteoporosis^[49]^. Valisena *et al* evaluated the management of high-energy blunt PRIs and found average ages of 44.2 years for hemodynamically stable patients and 46.6 years for unstable patients, with no significant difference between the two groups^[50]^. Typically, younger patients sustain these injuries through high-energy impacts, whereas older individuals experience them through low-energy impacts, such as falls^[51,52]^. The mechanisms underlying PRIs differ markedly between young and older adults. Younger patients often present with more complex fracture patterns that may alter anatomic structures and are associated with stronger muscles, which might necessitate more traction and retraction during surgery, resulting in larger wounds. Conversely, older individuals tend to have simpler fractures since their muscle tone is less robust, which relates to less surgical manipulation and smaller wounds. Additionally, nerve injury symptoms in older patients may be less pronounced, and their tolerance for such injuries could be higher.

## Limitations

This study had some limitations. First, the number of studies on certain groups was limited, and head-to-head comparisons within interventions were lacking. Although many single-arm studies reporting nerve injuries were excluded, the NMA provided direct and indirect evidence compared with traditional meta-analyses. Second, anterior and posterior fixation groups were not well-defined, as most studies focused on one or the other, potentially introducing bias. Third, the selection of studies was limited by inconsistent reporting of INIs as primary outcomes, highlighting a common issue in the literature review process. Heterogeneity existed across studies in terms of design, surgical techniques, devices used, and fracture severity; however, no evidence of reporting bias was detected. Although the low-to-moderate GRADE evidence rating might limit the application of our findings, our study primarily focused on complications arising from different surgical approaches. The variability in surgical techniques across the studies and the lack of a unified standard for classification limited detailed subgroup analyses for surgical approaches. Thus, further division based on detailed surgical techniques or constructs would result in excessive subgrouping, complicating data analysis and reducing statistical power. Additionally, a meta-regression analysis comparing randomized and non-randomized trials showed no statistically significant differences, indicating similar effects regardless of study design. Moreover, although differences in follow-up periods and advancements in surgical techniques over time could influence outcomes, our meta-regression analysis showed that publication year did not significantly moderate the results. Regarding the lack of long-term follow-up data, most included studies did not track the long-term outcomes of iatrogenic nerve injuries, and functional scores may not fully reflect these injuries as they are often influenced by multiple factors such as concomitant injuries, quality of fracture reduction, and other surgery-related complications. Last, this NMA focused solely on INIs, emphasizing their importance in PRI approaches. The choice of the operative method should balance functional outcomes with treatment efficacy and safety in clinical practice.

## Conclusion

Using NMA, we identified that the RA technique had the lowest INI incidence among PRI treatment methods. When feasible, the posterior approach results in lower INI risk compared with the anterior approach. Selecting the appropriate surgical approach is crucial to balance effective treatment with the risk of iatrogenic complications. Each approach has associated vulnerable nerves; therefore, surgeons should be aware of nerve anatomy and aim to avoid injury with both closed and open techniques.

## Data Availability

The datasets analyzed during the current study are derived entirely from publicly available sources. These sources include published articles and reports, which are cited within the manuscript.

## Research registration unique identifying number (UIN)

PROSPERO (CRD42024581218).
